# Assessing sensitivity to assumptions in mixed effects analyses of stepped-wedge trials

**DOI:** 10.1186/1745-6215-16-S2-O46

**Published:** 2015-11-16

**Authors:** Calum Davey, Jennifer A Thompson

**Affiliations:** 1 Department of Infectious Disease Epidemiology, London School of Hygiene and Tropical Medicine, London, UK; 2 MRC London Hub for Trials Methodology Research, MRC Clinical Trials Unit, University College London, London, UK

## Background

Stepped-wedge trials are used to evaluate the impact of interventions. Researchers often use mixed effects regression to estimate effects. This method includes within-cluster - non-randomised - comparisons that requires assumptions about the secular trends.

## Methods

We simulated data from stepped-wedge trials with different characteristics. We analysed these data using a within-step only approach and mixed effects regression, and evaluated their performance. The within-step only approach preserves randomisation by combining estimates of effect from within steps using a weighted average; we used non-parametric bootstrapping to generate inferential statistics. We introduced violations of the mixed effects model assumptions and investigated the effects on the two methods.

## Results

When the assumptions were met, the mixed effects method was more sensitive and specific than the within-step approach. Bias was introduced to the mixed effects results by interaction of the secular trend with the clusters, and with the intervention. The within-step approach remained unbiased even in extreme violations of these assumptions. Comparing the mixed effects estimate of effect with the within-step estimate helped identify violations of the assumptions.

## Discussion

We confirmed that mixed effects methods are more powerful than a within-step method when assumptions are met. Moderate to severe violations of assumptions led to bias, supporting the need for clear reporting standards and sensitivity analysis for stepped-wedge trials. Estimating the within-step effect can be useful for identifying bias.

## Conclusion

Within-step analyses that preserve the randomisation should be used as a diagnostic to assess the validity of common mixed effects methods for analysing stepped-wedge trials.

